# Catecholamine-Secreting Non-small Cell Lung Cancer Presenting With Hypertensive Crisis: A Report of a Rare Case

**DOI:** 10.7759/cureus.86027

**Published:** 2025-06-14

**Authors:** Anas Kartoumah, Omar Ali, Talal Alomar, Mojir Muhajir, Mohamad Horani

**Affiliations:** 1 Biomedical Sciences, University of South Florida, Tampa, USA; 2 Oncology, University of Arizona College of Medicine - Tucson, Tucson, USA; 3 Internal Medicine, Creighton University School of Medicine, Phoenix, USA; 4 Radiology, Midwestern University Arizona College of Osteopathic Medicine, Glendale, USA; 5 Internal Medicine, Chandler Regional Medical Center, Chandler, USA

**Keywords:** catecholamine-secreting tumor, neuroendocrine differentiation, non-small cell lung cancer, paraneoplastic syndrome, pulmonary hypertensive crisis

## Abstract

Ectopic catecholamine production by non-small cell lung cancer (NSCLC) is exceptionally rare and, to our knowledge, has not been previously reported in the literature. We present the case of a 68-year-old woman with advanced lung adenocarcinoma who presented in hypertensive crisis due to markedly elevated normetanephrine levels. Initial suspicion was for pheochromocytoma, but normal chromogranin A levels suggested a paraneoplastic phenomenon. Despite alpha-adrenergic blockade, the patient’s metastatic disease and refractory hypertension contributed to poor treatment tolerance, ultimately necessitating transition to hospice care. This case underscores the importance of considering atypical endocrine manifestations of NSCLC and highlights the challenges of managing advanced cancer complicated by paraneoplastic syndromes.

## Introduction

Non-small cell lung cancer (NSCLC) is the most common type of lung malignancy, accounting for approximately 85% of lung cancer diagnoses [1]. Although significant progress has been made in diagnostic strategies and targeted therapies, patients often present with advanced or metastatic disease, resulting in high morbidity and mortality rates. Paraneoplastic syndromes, defined as clinical syndromes not directly attributable to tumor invasion but rather to tumor-derived substances or immune-mediated mechanisms, can further complicate the clinical course of NSCLC [2]. Paraneoplastic syndromes associated with lung cancer can impair various organ functions and manifest as endocrine, neurologic, dermatologic, rheumatologic, hematologic, and ophthalmologic syndromes [3]. Common paraneoplastic phenomena in lung cancer include hypercalcemia, syndrome of inappropriate antidiuretic hormone (SIADH ), and Cushing syndrome [4].

Non-small cell lung cancer has rarely been associated with catecholamine secretion, which can mirror a pheochromocytoma. Excess catecholamines can precipitate severe, episodic hypertension, referred to as a hypertensive crisis, characterized by dangerously elevated blood pressures and end-organ dysfunction. Recognizing and managing paraneoplastic hypertensive crises are vital because such episodes may result in cardiovascular events, stroke, or other complications. Here, we present what we believe to be the first documented case of catecholamine-secreting NSCLC, culminating in life-threatening hypertensive crises and representing a clinically significant and exceptionally rare paraneoplastic phenomenon.

## Case presentation

A 68-year-old woman with a 40-pack-year smoking history presented to the emergency department with progressive left hip pain after a mechanical fall. Imaging confirmed a displaced left femoral neck fracture with features concerning for a pathological fracture. She was admitted for urgent orthopedic evaluation, and a CT scan was performed for further assessment.

Her preoperative evaluation revealed severe, uncontrolled hypertension, with systolic blood pressures exceeding 200 mmHg. Despite medical management, her hypertension remained difficult to control. She underwent left total hip arthroplasty and was subsequently referred for further oncologic and endocrine evaluation.

Diagnostic assessment

Given her uncontrolled hypertension, hyponatremia (serum sodium: 126 mmol/L), and history of smoking, further oncologic and endocrine workup was pursued. Key laboratory values are summarized in Table 1, and imaging and pathologic findings are detailed in Table 2.

**Table 1:** Key laboratory findings SIADH: Syndrome of inappropriate antidiuretic hormone

Test	Result	Reference range	Interpretation
Plasma normetanephrines	5.4 nmol/L	0.00-0.89 nmol/L	Markedly elevated
24-hour urinary normetanephrines	2,238 μg/g creatinine	0-400 μg/g creatinine	Markedly elevated
Chromogranin A	Normal	–	Argues against pheochromocytoma
Serum sodium	126 mmol/L	135-145 mmol/L	Hyponatremia, a potential SIADH indicator

**Table 2:** Imaging and pathologic findings NSCLC: Non-small cell lung cancer, Bx: Biopsy

Modality	Findings	Interpretation
CT chest/abdomen/pelvis	Multiple lung nodules, left renal mass, bilateral adrenal lesions, bone mets	Suggests widespread metastatic disease
Nuclear medicine bone scan	↑ Uptake in the humeral head, greater trochanter, and tibial metaphyses	Metastatic osseous involvement confirmed
MRI pelvis	4.4×3.2×2.6 cm lesion at right greater trochanter	Consistent with skeletal metastasis
Biopsy (hip lesion)	Adenocarcinoma consistent with lung origin	Primary NSCLC confirmed
Bronchoscopic lymph node Bx	Positive for malignant cells	Supported metastatic NSCLC diagnosis

**Figure 1:**
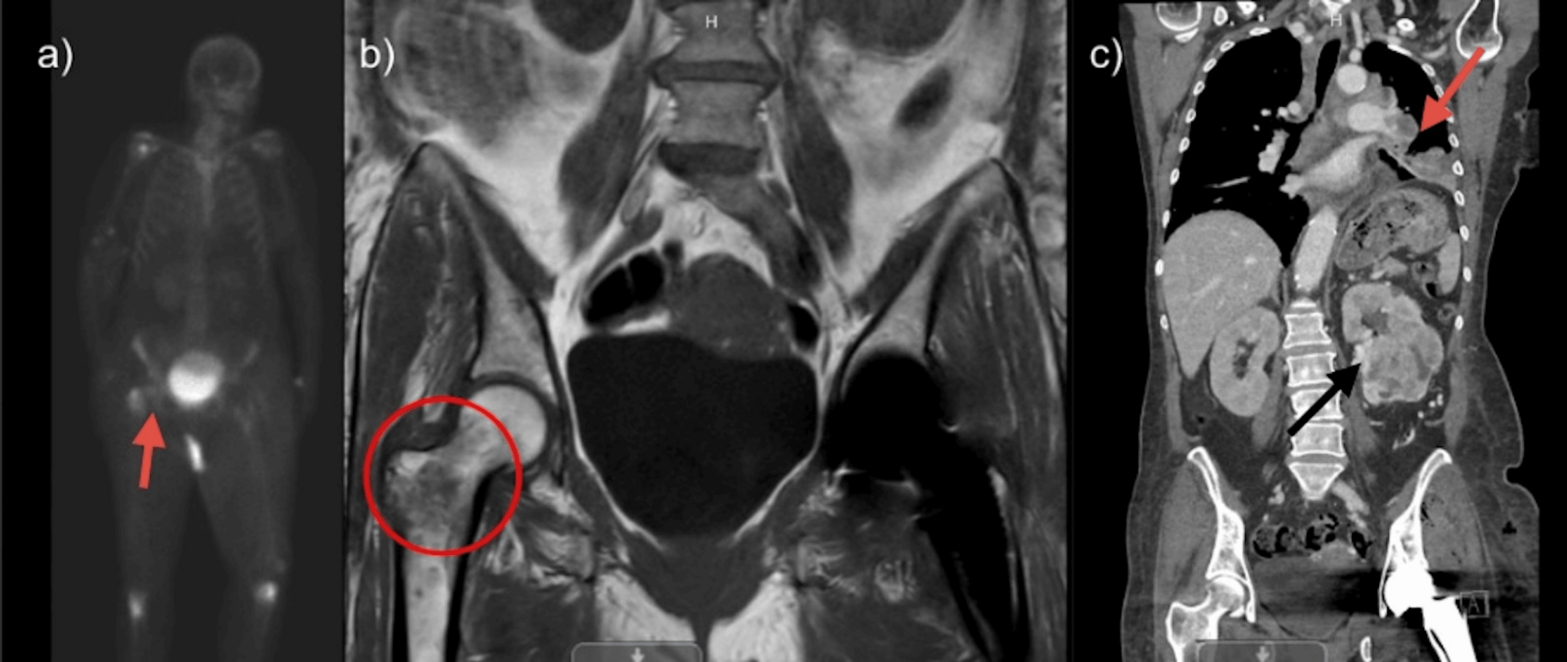
Imaging findings a) Nuclear medicine bone imaging with technetium-99 show increased uptake (red arrow) in the right humeral head, right greater trochanter of the femur, and bilateral tibial metaphyses suggestive of metastatic disease; b) MRI of the pelvis showing a 4.4x3.2x2.6 cm lesion of the right greater trochanter circled in red; c) CT scan showing large left renal mass and left lung masses (red arrow)

Given the concerning imaging findings, a nuclear medicine bone scan and MRI of the pelvis were also obtained, as bone metastases are common in advanced lung cancer and often require biomarkers such as NTx and CTx for prognostic assessment [5]. Metastatic lesions were confirmed on multimodal imaging (Figure 1). Biopsy of the hip lesion confirmed adenocarcinoma consistent with primary lung origin. Bronchoscopic lymph node sampling further supported the NSCLC diagnosis.

Treatment

The patient was initiated on alpha-blockade with phenoxybenzamine (starting dose 10 mg twice daily, titrated to 20 mg twice daily) to manage hypertensive crises. Beta-blockers were avoided initially to prevent unopposed alpha-adrenergic stimulation, which could exacerbate hypertension [6]. Despite alpha-blockade, hypertension remained refractory, requiring additional antihypertensives. The full treatment protocol is summarized in Table 3. However, chemotherapy was poorly tolerated, leading to significant fatigue and worsening functional status.

**Table 3:** Treatment summary NSCLC: Non-small cell lung cancer, BID: Twice a day, ACE: Angiotensin-converting enzyme, AUC: Area under the curve

Intervention	Agent/Procedure	Details/Dosage	Response/Outcome
Hypertension management	Alpha-blocker (phenoxybenzamine)	10 mg BID → titrated to 20 mg BID	Partial control; hypertensive crises persisted
	Calcium channel blocker (amlodipine)	10 mg daily	Adjunctive antihypertensive
	ACE inhibitor (lisinopril)	5 mg → 10 mg daily	Limited effect
NSCLC chemotherapy	Carboplatin + pemetrexed	AUC 5 + 500 mg/m² IV on Day 1	Poorly tolerated; functional decline
End-of-life transition	Hospice care	Home-based, due to progressive disease and poor tolerance	Patient died ~2 months post-diagnosis

Outcome and follow-up

Following one cycle of chemotherapy, the patient experienced severe intolerance, leading to a deterioration in performance status. Her hypertension remained poorly controlled, and she developed progressive functional decline. Due to limited treatment options and poor prognosis, the patient was transitioned to home hospice care. She remained hemodynamically unstable, with persistent hypertensive crises despite maximal medical therapy [7]. She ultimately died due to complications of advanced disease, including progressive multi-organ failure.

## Discussion

Paraneoplastic syndromes occur when tumors secrete hormones, cytokines, or biologically active substances, or stimulate immune responses that result in clinical manifestations distinct from direct tumor invasion [8]. These syndromes are particularly well-documented in small cell lung cancer (SCLC), where both endocrine and neurologic manifestations are frequently observed. In fact, studies have highlighted the strong association between SCLC and various paraneoplastic syndromes, emphasizing their role in early cancer detection and prognosis [9]. The incidence of paraneoplastic syndrome among patients with NSCLC has been reported to be significantly higher than in the general population, with a nearly fivefold greater incidence rate, underscoring the importance of recognizing these syndromes in clinical practice [10].

Lung cancers, especially SCLC, are notorious for producing ectopic hormones such as adrenocorticotropic hormone (ACTH) and antidiuretic hormone (ADH) [11]. In rare cases, ACTH-producing pheochromocytomas or paragangliomas can also contribute to ectopic Cushing’s syndrome, underscoring the complexity of endocrine manifestations in malignancies [12]. In contrast, catecholamine secretion in lung cancer is exceedingly rare. A comprehensive literature search revealed no previously reported cases of ectopic catecholamine production by NSCLC, to the best of our knowledge [13]. However, limitations include the lack of tissue-level catecholamine staining to confirm tumor secretion and the potential for confounding by bilateral adrenal lesions. This underscores the exceptional nature of our case and highlights the importance of considering paraneoplastic catecholamine secretion in the diagnosis when patients with NSCLC present with hypertensive crises, despite its rarity.

Catecholamines (including epinephrine, norepinephrine, and their metabolites) are primarily produced in the adrenal medulla or extra-adrenal paraganglia. Tumors that contain neuroendocrine features, such as pheochromocytomas, can secrete high levels of these substances, leading to episodic or persistent hypertension, sweating, palpitations, and headaches. In rare instances, adenocarcinomas or other NSCLC subtypes may acquire neuroendocrine-like capabilities and secrete catecholamines, mimicking a pheochromocytoma [14].

When a hypertensive crisis arises in a patient with newly discovered adrenal or lung lesions, the default suspicion often points toward pheochromocytoma. Testing for plasma-free metanephrines or fractionated metanephrines in 24-hour urine is highly sensitive for pheochromocytoma, and chromogranin A is frequently elevated in most catecholamine-producing tumors, boasting a negative predictive value of up to 95% [15]. However, normal chromogranin A levels in this scenario prompted reconsideration of the diagnosis, suggesting a paraneoplastic cause unrelated to a classic pheochromocytoma. Follow-up imaging of the chest clarified that the source of the ectopic hormone production likely originated from the lung tumor, rather than a primary adrenal neoplasm.

Persistent, severe hypertension may precipitate end-organ damage, including hemorrhagic or ischemic stroke, acute heart failure, and aortic dissection-risks amplified in individuals with significant cardiovascular comorbidities, such as a cerebral aneurysm. Adequate alpha-blockade (with or without concurrent beta-blockade) is essential to minimize the potentially fatal consequences of catecholamine surges [8]. However, clinicians often struggle to control paraneoplastic catecholamine secretion unless the underlying malignancy is aggressively managed, highlighting the importance of multidisciplinary diagnostic and therapeutic strategies.

Standard treatments for advanced NSCLC include systemic chemotherapy, molecularly targeted therapies (e.g., estimated glomerular filtration rate (EGFR) or anaplastic lymphoma kinase (ALK) inhibitors), and immunotherapy [16]. In patients burdened by extensive metastatic disease and heavy comorbidities, these options may be poorly tolerated or ineffective. Although alpha-blockers and other antihypertensives can provide symptomatic relief, definitive control of ectopic hormone production often requires successful tumor debulking or therapies that induce sufficient tumor regression.

Additionally, older patients or those with multiple comorbidities must contend with the malignant process as well as cardiovascular instability triggered by excess catecholamines. This instability can worsen existing conditions, such as coronary artery disease or cerebrovascular disease, further limiting therapeutic choices [17]. Even modern targeted therapies or immunotherapies may fail when tumor burden is extensive or treatment toxicities prove intolerable, underscoring the complexity of care in such cases.

Paraneoplastic hypertensive crises can be life-threatening, and survival ultimately depends on tumor biology, treatment tolerability, and response to cancer-directed therapies. In advanced-stage NSCLC with limited therapeutic options, outcomes can be dismal, even with supportive medical interventions [18]. This case highlights the difficulties of diagnosing and managing rare paraneoplastic syndromes when the malignancy is widespread, emphasizing the importance of early recognition and a multidisciplinary approach.

Learning points

Catecholamine-secreting NSCLC represents a previously undocumented paraneoplastic syndrome. Measurement of plasma metanephrines and chromogranin A levels aids in distinguishing NSCLC-related catecholamine secretion from pheochromocytoma. Refractory hypertensive crises in NSCLC should prompt consideration of ectopic catecholamine production. Management is challenging due to poor treatment tolerance in advanced disease.

## Conclusions

To our knowledge, this is the first documented case of catecholamine-secreting NSCLC, representing a rare and clinically significant paraneoplastic phenomenon. Early recognition of such atypical endocrine manifestations is essential for prompt diagnostic workup and appropriate management. While pheochromocytoma is often suspected in the setting of hypertensive crises and elevated catecholamines, clinicians should consider alternative sources in the presence of widespread malignancy and normal chromogranin A levels.

Clinicians who encounter refractory or episodic hypertension in patients with known or suspected NSCLC should obtain plasma metanephrines and chromogranin A levels early, begin alpha-adrenergic blockade before any beta-blocker, and expedite oncologic staging to guide tumor-directed therapy. Multidisciplinary management involving oncology, endocrinology, cardiology, and palliative care teams can optimize blood pressure control and treatment tolerance. Our conclusions are limited by the single-patient design, the absence of tumor immunohistochemistry for catecholamines, and possible confounding from adrenal lesions. Additional cases or prospective registries are needed to confirm these findings and refine treatment algorithms.
